# Regional disparities and risk factors of mortality among patients at high risk of sudden cardiac death in emerging countries: a nonrandomized controlled trial

**DOI:** 10.1186/s12916-024-03310-5

**Published:** 2024-03-22

**Authors:** Shuang Zhao, Chi-Keong Ching, Dejia Huang, Yen-Bin Liu, Diego A. Rodriguez-Guerrero, Azlan Hussin, Young-Hoon Kim, Brian Van Dorn, Xiaohong Zhou, Balbir Singh, Shu Zhang, the Improve SCA Investigators

**Affiliations:** 1 State Key Laboratory of Cardiovascular Disease, Arrhythmia Center, Fuwai Hospital, National Center for Cardiovascular Diseases, Chinese Academy of Medical Sciences and Peking Union Medical College, No.167 Beilishi Rd, Xicheng District, Beijing, 100037 China; 2 National Heart Centre Singapore, Outram District, Singapore, Singapore; 3 West China Hospital, Chengdu, China; 4 National Taiwan University Hospital, Taipei City, Taiwan China; 5 Instituto de Cardiología Fundación Cardioinfantil, Centro Internacional de Arritmias, Bogotá, Colombia; 6 Universidad de La Sabana, Bogota, Colombia; 7 Institut Jantung Negara, Kuala Lumpur, Malaysia; 8 Korea University Medical Center, Seoul, Republic of Korea; 9 Medtronic Inc., Mounds View, MN USA; 10 Pan Max Hospital, Delhi, India; 11 Max Super Speciality Hospital, Delhi, India

**Keywords:** Sudden cardiac death, Mortality, Implantable cardioverter-defibrillator, Emerging countries, Risk factor, Asian

## Abstract

**Background:**

Comprehensive data on patients at high risk of sudden cardiac death (SCD) in emerging countries are lacking. The aim was to deepen our understanding of the SCD phenotype and identify risk factors for death among patients at high risk of SCD in emerging countries.

**Methods:**

Patients who met the class I indication for implantable cardioverter-defibrillator (ICD) implantation according to guideline recommendations in 17 countries and regions underrepresented in previous trials were enrolled. Countries were stratified by the WHO regional classification. Patients were or were not implanted with an ICD at their discretion. The outcomes were all-cause mortality and SCD.

**Results:**

We enrolled 4222 patients, and 3889 patients were included in the analysis. The mean follow-up period was 21.6 ± 10.2 months. There were 433 (11.1%) instances of all-cause mortality and 117 (3.0%) cases of SCD. All-cause mortality was highest in primary prevention (PP) patients from Southeast Asia and secondary prevention (SP) patients from the Middle East and Africa. The SCD rates among PP and SP patients were both highest in South Asia. Multivariate Cox regression modelling demonstrated that in addition to the independent predictors identified in previous studies, both geographic region and ICD use were associated with all-cause mortality in patients with high SCD risk. Primary prophylactic ICD implantation was associated with a 36% (HR = 0.64, 95% CI 0.531–0.802, *p* < 0.0001) lower all-cause mortality risk and an 80% (HR = 0.20, 95% CI = 0.116–0.343, *p* < 0.0001) lower SCD risk.

**Conclusions:**

There was significant heterogeneity among patients with high SCD risk in emerging countries. The influences of geographic regions on patient characteristics and outcomes were significant. Improvement in increasing ICD utilization and uptake of guideline-directed medical therapy in emerging countries is urgent.

**Trial registration:**

ClinicalTrials.gov, NCT02099721.

**Supplementary Information:**

The online version contains supplementary material available at 10.1186/s12916-024-03310-5.

## Background

Sudden cardiac death (SCD) remains a leading cause of cardiovascular mortality worldwide. The burden of SCD varies by geographic region, although global data are somewhat limited. In developed countries, such as the United States of America (USA) and the Netherlands, SCD incidence rate estimates range from 53 to 117 per 100,000 persons per year, while in emerging countries, such as China and India, SCD incidence rate estimates range from 41.8 to 181 per 100,000 persons per year [1, 2]. Data indicate that various causes, risks and predisposing conditions vary in prevalence according to factors such as geographic region and ethnicity, influencing the incidence of SCD [3]. However, comprehensive data on patients with high SCD risk in emerging countries are lacking, and disease management strategies and resources vary substantially by geographic region.

There is substantial evidence in favour of implantable cardioverter-defibrillators (ICDs) as therapy for the prevention of SCD. However, ICD utilization remains low in emerging countries, especially in primary prevention (PP) patients [4]. In 2017, the ICD implantation rates per million persons in China and India were 2.9 and 2.6, respectively, while the rate was 280 per million persons in the USA [5]. Previous evidence supporting ICD utilization as a therapy to prevent SCD came from developed, high-income regions such as North America and Europe, with little information from other geographic regions. Thus, the benefits of ICD implantation in emerging countries need to be further demonstrated.

In this sub-analysis of the Improve Sudden Cardiac Arrest (SCA) study, we aimed to deepen our understanding of the SCD phenotype in emerging countries, identify risk factors for death and guide the allocation of medical resources in emerging countries where data are limited. In addition, we analysed differences between the Improve SCA study and previous large-scale clinical trials conducted in developed countries.

## Methods

### Study design and participants

The detailed methods of the Improve SCA study have been previously published [6, 7]. In brief, the Improve SCA study (ClinicalTrials.gov ID: NCT02099721) was a prospective, nonrandomized, nonblinded, multicentre global, postmarket study. Key inclusion criteria were patients who met the class I indication for the implantation of an ICD/cardiac resynchronization therapy-defibrillator (CRT-D) according to the American College of Cardiology/American Heart Association/Heart Rhythm Society (ACC/AHA/HRS) or European Society of Cardiology (ESC) guideline recommendations. All enrolled subjects were required to meet the primary or secondary guidelines for implantation. The decision to implant an ICD (or CRT-D, if indicated) was left to the discretion of the patient and the physician. Key exclusion criteria were patients aged ≤ 18 years who had any ICD/CRT-D contraindications. The Improve SCA study was conducted from March 2014 to August 2018 at 86 sites in 17 countries and regions underrepresented in prior randomized clinical trials and where ICD (or CRT-D) utilization in clinically indicated patients is low: East Asia, Southeast Asia, South Asia, Latin America, Eastern Europe, the Middle East and Africa.

The investigation conformed with the principles outlined in the Declaration of Helsinki.

### Outcomes

The outcomes were the time to all-cause mortality and SCD. SCD was defined as natural death due to cardiac causes, indicated by the abrupt loss of consciousness within 1 h of the onset of acute symptoms; the presence of preexisting heart disease may have been known, but the time and mode of death were unexpected. If the time of onset could not be determined, SCD was alternatively defined as any unexpected cardiac death occurring out of the hospital or in the emergency room indicated as dead on arrival.

### Data collection

Clinical data were collected at designated time points throughout the study using an electronic data management system. Data were anonymized and stored in a secure, password-protected database that was backed up daily. Data were reviewed using programmed and manual data checks, and data queries were made available to study sites for resolution.

### Statistical analyses

Standard descriptive statistics were used to describe baseline characteristics in the overall cohort and by geographic region and country income classification. For comparisons between groups, one-way analysis of variance was used for all variables. Kaplan–Meier survival curves were used to evaluate survival time according to geographic region. Univariate and multivariate Cox proportional hazards models for time-to-event analysis of all-cause mortality in PP and ICD (or CRT-D) implanted SP patients were used. For all-cause mortality, 95% confidence intervals (CIs) were calculated for the hazard ratios for each variable. To create the multivariate Cox proportional hazards model, backward regression was performed, initially incorporating all variables analysed in the univariate analysis but ultimately retaining age, New York Heart Association (NYHA) classification, ischaemic cardiomyopathy (ICM) and any other variables that were significant at the 0.05 level after the nonsignificant variables were removed from the multivariate model. Age, NYHA classification and ICM were included in the multivariate analyses regardless of significance based on known strong associations with mortality.

Countries were stratified by WHO regional classification based on the World Bank classification system. Groups were defined as follows:

1. *Geographic regions*:
*East Asia* (*n* = 2396)—China, China-Taiwan, Korea; *Southeast Asia* (*n* = 285)—Singapore, Malaysia; *South Asia* (*n* = 813)—India; *Latin America* (*n* = 177)—Argentina, Brazil, Colombia, Mexico; *Europe* (*n* = 110)—Belarus, Malta, Russian Federation; and *the Middle East and Africa* (*n* = 108)—Egypt, Tunisia, United Arab Emirates, South Africa.
2. *Income level classification*:
  Countries were grouped into regions using the World Bank classification system updated in 2014 (http://dataworldbankorg/about/country-and-lending-groups):
*Lower middle income (LMI)* (*n* = 825): US$1046–$4125. India, Egypt.
*Upper middle income (UMI)* (*n* = 1948): US$4126–$12,735. China, Malaysia, Brazil, Colombia, Mexico, Belarus, Tunisia, South Africa.
*High income (HI)* (*n* = 1116): > US$12,735. China-Taiwan, Korea, Singapore, Argentina, Malta, Russian Federation, United Arab Emirates.

## Results

Between March 2014 and July 2017, 4222 patients were enrolled with a mean follow-up of 21.6 ± 10.2 months. A total of 3889 patients were included in the analysis presented here with 333 patients excluded. There were 2696 (69.3%) PP patients and 1193 (30.7%) secondary prevention (SP) patients, and the percentages of PP and SP patients implanted with ICDs (or CRT-Ds) were 51.9% and 89.4%, respectively.

### Heterogeneity among patients with high SCD risk in emerging countries by region

**Fig. 1:**
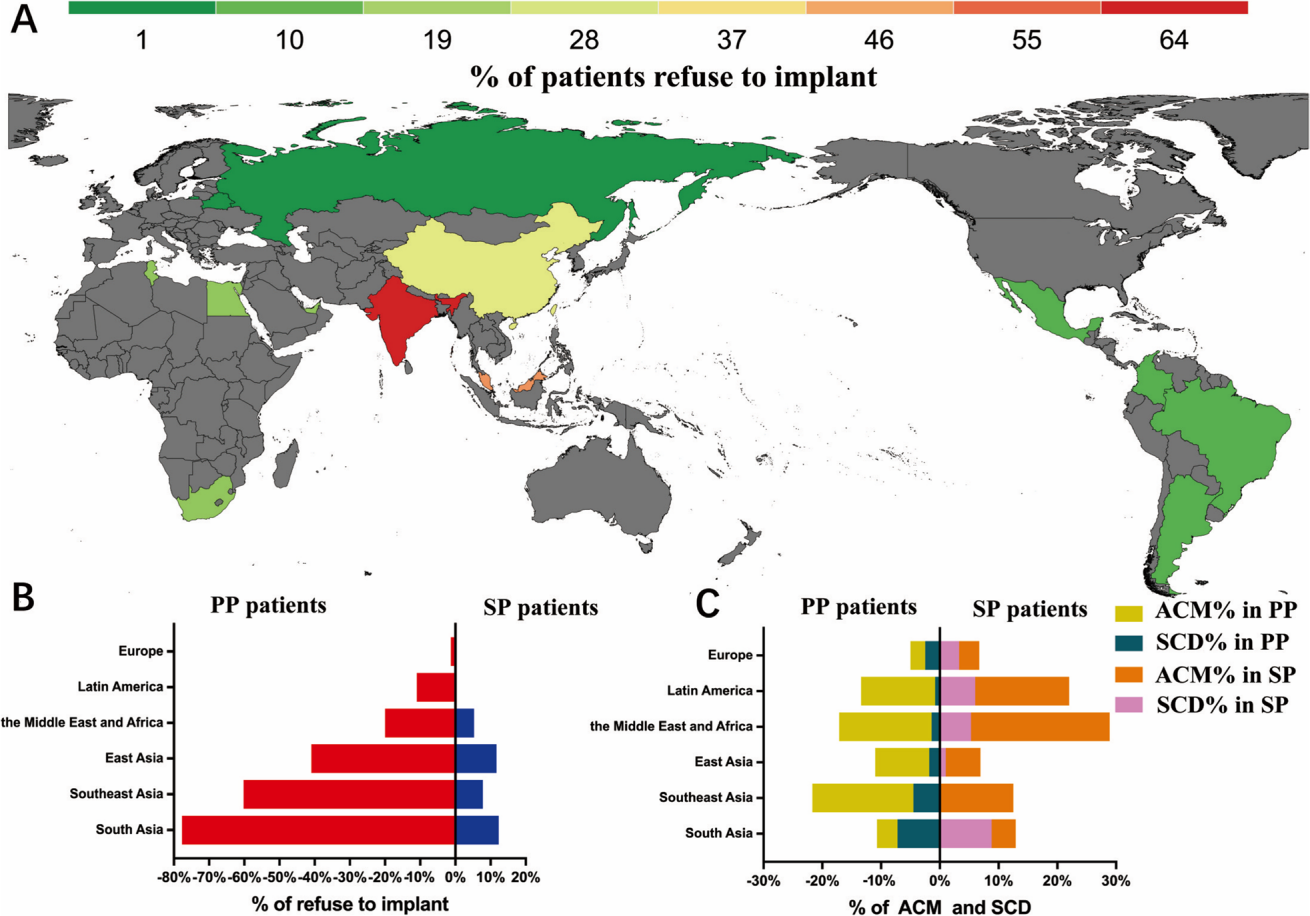
Graphical display of implantation by region. **a** World map showing the percentage of patients without ICDs/CRT-Ds. **b** Percentage of PP and SP patients without ICDs/CRT-Ds. **c** Percentage of PP and SP patients with all-cause mortality and SCD. Abbreviations: ACM, all-cause mortality; ICD, implantable cardioverter-defibrillator; CRT-D, cardiac resynchronization therapy-defibrillator; PP, primary prevention; SP, secondary prevention; SCD, sudden cardiac death

Overall, patient demographics, ICD (or CRT-D) indications, medical histories and medications varied widely by geographic region (Table 1). The mean age was 59.0 ± 13.3 years old, and 76.7% of the patients were male. Significant heterogeneity in age (*p* < 0.0001) was observed across the six geographic regions. Patients from South Asia had the youngest mean age at 56.2 ± 12.4 years old, while patients from Latin America had the oldest mean age (61.8 ± 13.2 years old). The comorbidity burden also showed strong regional variations (*p* < 0.0001), with the highest prevalence of hypertension (67.0%) and diabetes (46.8%) in Southeast Asia; the prevalence of hypertension was the lowest in the Middle East and Africa (27.8%), and the prevalence of diabetes was the lowest in Europe (15.5%). Non-ischaemic cardiomyopathy (NICM) occurred most frequently in East Asia (63.4%) and least frequently in Europe (44.5%). ICM occurred most frequently in Latin America (47.5%) and least frequently in East Asia (16.4%). The mean left ventricular ejection fraction (LVEF) was higher in patients in Europe and East Asia (33.7 ± 13.0% and 33.7 ± 13.6%, respectively) than in Southeast Asia (25.8 ± 9.5%). Regional differences in the use of medication and ICD (or CRT-D) implantation rates were also observed. The percentage of patients taking beta blockers was highest in the Middle East and Africa (81.5%), while the least was in East Asia (68.7%). The percentage of patients taking angiotensin-converting enzyme inhibitors/angiotensin II receptor blockers (ACEIs/ARBs) was high in Europe (83.6%), while the least was in South Asia and Southeast Asia (60.4%). Although the smallest number of PP patients was found in the Middle East and Africa (64.8%) and the largest number of PP patients was found in South Asia (79.0%), the percentage of patients taking beta blockers and ACEIs/ARBs was greater in the Middle East and Africa than in South Asia (81.5% vs. 76.6%, 79.6% vs. 60.4%). The percentage of PP patients with implants was highest in Europe (98.8%), and the percentages of SP patients with implants were highest in Europe (100%) and Latin America (100%) (Fig. 1). The percentage of patients without ICDs (or CRT-Ds) in the indicated patients by geographic region is shown in Fig. 1. The percentages of PP and SP patients without ICDs/CRT-Ds were highest in South Asia (77.7% and 12.3%, respectively), and these values were the lowest in Europe (1.2% and 0%) (Fig. 1). The percentages of PP and SP patients with SCD were highest in South Asia (7.2% and 8.8%, respectively) (Fig. 1c). The *p*-values for all of the above regional comparisons were < 0.0001.

**Table 1:** Baseline demographic and clinical characteristics by geographic region

**Characteristics**	**Overall**	**Geographical region**						
		**East Asia**	**South Asia**	**Southeast** **Asia**	**Latin America**	**Europe**	**The Middle East and Africa**	***P*** ** value**
***N***	3889	2396	813	285	177	110	108	
**Demographics**								
Age (years)	59.0 ± 13.3	59.5 ± 13.7	56.2 ± 12.4	61.2 ± 11.4	61.8 ± 13.2	58.8 ± 9.7	59.8 ± 14.6	< .0001
Male	2981 (76.7)	1771 (73.9)	652 (80.2)	240 (84.2)	134 (75.7)	89 (80.9)	95 (88.0)	< .0001
**Indication for ICD/CRT-D**								
PP	2696 (69.3)	1556 (64.9)	642 (79.0)	221 (77.5)	127 (71.8)	80 (72.7)	70 (64.8)	< .0001
ICD/CRT-D implant	2465 (63.4)	1662 (69.4)	292 (35.9)	147 (51.6)	163 (92.1)	109 (99.1)	94 (87.0)	< .0001
CRT-D implanted	811 (20.9)	535 (22.3)	89 (10.9)	24 (8.4)	64 (36.2)	58 (52.7)	41 (38.0)	< .0001
**Medical history**								
NYHA Class, I/II/III/IV (%)	5.1/43.4/ 42.8/1.1	5/30.3/53.4/1.5	4.2/68.5/23.2/0.5	6/76.8/11.6/0	10.7/52.5/31.6/0.6	2.7/33.6/61.8/0	6.5/50/38/1.9	< .0001
Coronary artery disease	1660 (42.7)	818 (34.1)	463 (57.0)	202 (70.9)	73 (41.2)	58 (52.7)	46 (42.6)	< .0001
ICM	838 (21.5)	392 (16.4)	223 (27.4)	67 (23.5)	84 (47.5)	31 (28.2)	41 (38.0)	< .0001
NICM	2238 (57.5)	1518 (63.4)	382 (47.0)	150 (52.6)	82 (46.3)	49 (44.5)	57 (52.8)	< .0001
Dilated cardiomyopathy	2119 (54.5)	1414 (59.0)	363 (44.6)	147 (51.6)	87 (49.2)	47 (42.7)	61 (56.5)	< .0001
Hypertrophic Cardiomyopathy	92 (2.4)	78 (3.3)	8 (1.0)	1 (0.4)	3 (1.7)	1 (0.9)	1 (0.9)	0.0005
Valvular disfunction	1070 (27.5)	804 (33.6)	94 (11.6)	44 (15.4)	63 (35.6)	52 (47.3)	13 (12.0)	< .0001
Primary/idiopathic electrical disease	160 (4.1)	118 (4.9)	12 (1.5)	7 (2.5)	19 (10.7)	1 (0.9)	3 (2.8)	< .0001
Idiopathic structural heart disease	55 (1.4)	20 (0.8)	11 (1.4)	0 (0.0)	15 (8.5)	3 (2.7)	5 (5.6)	< .0001
Congestive heart failure	1323 (34)	929 (38.8)	121 (14.9)	139 (48.8)	45 (25.4)	60 (54.5)	29 (26.9)	< .0001
Syncope or presyncope	672 (17.3)	426 (17.8)	117 (14.4)	25 (8.8)	38 (21.5)	23 (20.9)	43 (39.8)	< .0001
NSVT	986 (25.4)	745 (31.1)	92 (11.3)	39 (13.7)	42 (23.7)	38 (34.5)	30 (27.8)	< .0001
Hypertension	1436 (36.9)	843 (35.2)	251 (30.9)	191 (67.0)	60 (33.9)	61 (55.5)	30 (27.8)	< .0001
Diabetes	1066 (27.7)	518 (21.7)	317 (40.7)	133 (46.8)	40 (22.6)	17 (15.5)	41 (38.0)	< .0001
Myocardial infarction	1260 (32.4)	544 (22.7)	423 (52.0)	123 (43.2)	81 (45.8)	48 (43.6)	41 (38.0)	< .0001
LBBB	735 (18.9)	410 (17.1)	169 (20.8)	21 (7.4)	45 (25.4)	48 (43.6)	42 (38.9)	< .0001
PR duration (ms)	175 ± 41.1	178 ± 41.3	164.8 ± 37.9	179.9 ± 36.9	169.1 ± 46.5	188.4 ± 37.2	170.4 ± 48.7	< .0001
QRS duration (ms)	121.7 + 34.3	122.7 ± 34.3	119.3 ± 35.2	108.3 ± 23.5	125.3 ± 37.1	139.0 ± 37.7	128.6 ± 33.6	< .0001
LVEF (%)	31.6 ± 12.5	33.7 ± 13.6	28.2 ± 7.8	25.8 ± 9.5	28.7 ± 11.1	33.7 ± 13.0	29.1 ± 11.1	< .0001
**Baseline therapy**								
Antiarrhythmics, excluding beta blockers	1660 (42.7)	1162 (48.5)	261 (32.1)	56 (19.6)	95 (53.7)	43 (39.1)	43 (39.8)	< .0001
Beta blockers	2772 (71.35)	1645 (68.7)	623 (76.6)	207 (72.6)	124 (70.1)	85 (77.3)	88 (81.5)	< .0001
ACEI/ARB	2467 (63.4)	1500 (62.6)	491 (60.4)	172 (60.4)	126 (71.2)	92 (83.6)	86 (79.6)	< .0001
Diuretics	2793 (71.8)	1702 (71.0)	607 (74.7)	178 (62.5)	124 (70.1)	92 (83.6)	90 (83.3)	< .0001
**Outcomes**								
All-cause mortality (PP)	321 (11.9)	171 (11.0)	69 (10.7)	48 (21.7)	17 (13.4)	4 (5.0)	12 (17.1)	< .0001
All-cause mortality (SP)	112 (9.4)	58 (6.9)	22 (12.9)	8 (12.5)	11 (22.0)	2 (6.7)	11 (28.9)	< .0001
SCD (PP)	88 (3.3)	28 (1.8)	46 (7.2)	10 (4.5)	1 (0.8)	2 (2.5)	1 (1.4)	< .0001
SCD (SP)	29 (2.4)	8 (1.0)	15 (8.8)	0 (0.0)	3 (6.0)	1 (3.3)	2 (5.3)	< .0001

*Abbreviations: PP* primary prevention, *ICD* implantable cardioverter-defibrillator, *CRT-D* cardiac resynchronization therapy-defibrillator, *NYHA* New York Heart Association, *ICM* ischaemic cardiomyopathy, *NICM* non-ischaemic cardiomyopathy, *NSVT* non-sustained ventricular tachycardia, *LBBB* left Buddle Branch Block, *LVEF* left ventricular ejection fraction, *ACEI/ARB* angiotensin-converting enzyme inhibitor/angiotensin receptor blocker, *SP* secondary prevention, *SCD* sudden cardiac death

### All-cause mortality and SCD among patients with high SCD risk in emerging countries

**Fig. 2:**
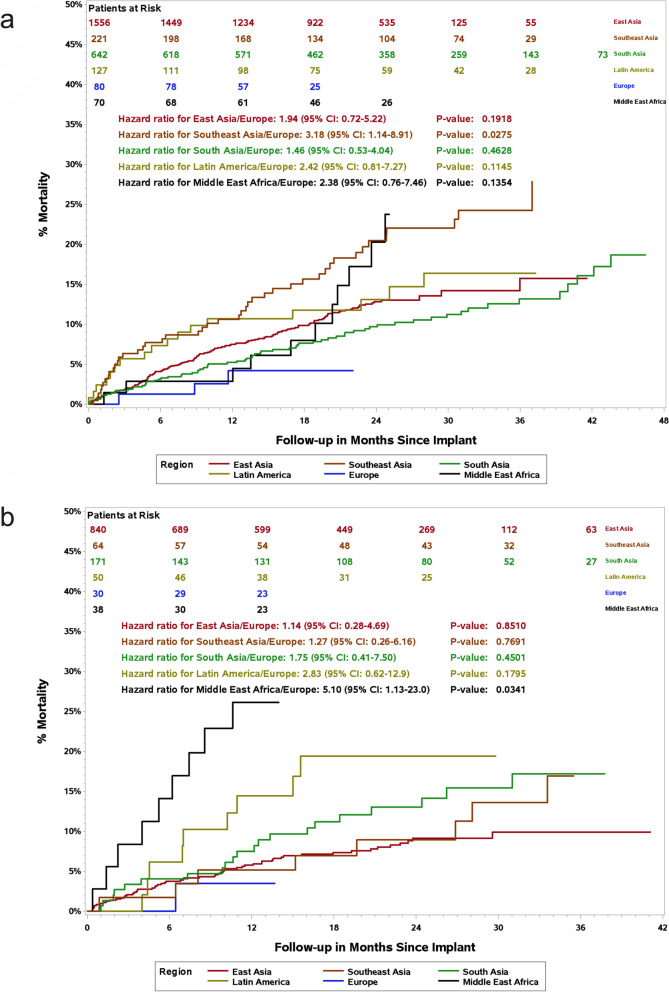
Kaplan–Meier curves showing the all-cause mortality rate among PP patients (**a**) and SP patients (**b**) stratified by geographic region. Abbreviations: PP, primary prevention; SP, secondary prevention

Of the 3889 patients, 433 (11.1%) died, including 321 (11.9%) PP patients and 112 (9.4%) SP patients. Patients from Europe had the lowest all-cause mortality rate (6 [5.5%] of 110), and those from the Middle East and Africa had the highest (23 [21.3%] of 108) (Table 1). Figure 2 shows Kaplan–Meier survival curves of all-cause mortality stratified by geographic region. All-cause mortality rates among PP and SP patients in the European region were the lowest, and the all-cause mortality rate in Southeast Asia was the highest among PP patients, which was significantly higher than that in Europe (hazard ratio [HR] = 3.18, 95% CI = 1.14–8.91, *p* = 0.0275). The all-cause mortality rate was the highest among SP patients from the Middle East and Africa (Middle East and Africa/Europe, HR = 5.1, 95% CI 1.13–23.0, *P* = 0.0341). The SCD rates were the highest among PP (46 [7.2%] of 642) and SP (15 [8.8%] of 171) patients from South Asia (Table 1). Patients’ demographics, clinical characteristics, the outcome of all-cause mortality and SCD by country income level are shown in Additional file 1: Table S1. The percentage of ICD/CRT-D-implanted patients was lower in the LMI countries than in the UMI and HI countries (36.4% vs. 70.6%, 76.3%, *p* < 0.0001). Patients from HI countries had the lowest all-cause mortality rate (104 [9.3%] of 1116) when compared with those from LMI and UMI countries (93 [11.3%] of 825 and 232 [11.9%] of 1948). The SCD rate of PP (46 [7.1%] of 646) and SP (15 [8.4%] of 179) patients of LMI countries was higher than that in UMI and HI countries (*p* < 0.0001).

### Risk factors associated with all-cause mortality among patients with high SCD risk in emerging countries

Table 2 shows the results of univariate and multivariate analyses of variance from a proportional hazards regression model of all-cause mortality in PP patients and SP patients with ICDs/CRT-Ds. After adjusting for the other variables in the model, the variables associated with all-cause mortality in PP patients included age, NYHA classification III-IV, congestive heart failure, diabetes, ICDs, CRT-Ds, a low LVEF, antiarrhythmic medication use (excluding beta blockers), a lack of ACEI/ARB use and whether the patient was from East Asia. For ICDs/CRT-Ds implanted SP patients, after adjusting for other model variables, variables associated with all-cause mortality included age, congestive heart failure, myocardial infarction, low LVEF, diuretic use, a lack of ACEI/ARB use and whether the patient was from Latin America, the Middle East and Africa.

**Table 2:** Univariate and multivariate analysis of variables associated with all-cause mortality in PP patients and SP patients with ICDs/CRT-Ds

	**Primary prevention patients**				**ICDs/CRT-Ds implanted secondary prevention patients**			
	**Univariate**		**Multivariate**		**Univariate**		**Multivariate**	
**Characteristics**	**Hazard ratio**	***P*** **-value**	**Hazard ratio**	***P*** **-value**	**Hazard ratio**	***P*** **-value**	**Hazard ratio**	***P*** **-value**
**Demographics**								
Age (years)	1.023 (1.013, 1.032)	< .0001	1.025 (1.015, 1.035)	< .0001	1.046 (1.031, 1.062)	< .0001	1.031 (1.014, 1.048)	0.0004
Gender male	0.886 (0.690, 1.139)	0.3455	0.897 (0.691, 1.163)	0.4107	1.708 (1.019, 2.863)	0.0421	1.497 (0.855, 2.621)	0.1583
**Medical History**								
NYHA III-IV	1.664 (1.326, 2.089)	< .0001	1.726 (1.322, 2.253)	< .0001	2.801 (1.930, 4.066)	< .0001	1.410 (0.918, 2.166)	0.1168
ICM	1.200 (0.931, 1.548)	0.1588	1.219 (0.928, 1.602)	0.1556	2.156 (1.468, 3.167)	< .0001	1.052 (0.679, 1.631)	0.8199
NICM	0.834 (0.664, 1.048)	0.1197			1.058 (0.714, 1.570)	0.7777		
Congestive heart failure	1.695 (1.361, 2.110)	< .0001	1.569 (1.246, 1.977)	0.0001	3.436 (2.357, 5.009)	< .0001	2.227 (1.466, 3.382)	0.0002
Syncope	0.881 (0.554, 1.401)	0.5923			1.153 (0.795, 1.671)	0.4536		
NSVT	1.313 (1.041, 1.656)	0.0214			0.815 (0.473, 1.404)	0.4614		
Hypertension	1.047 (0.837, 1.309)	0.6878			1.563 (1.076, 2.270)	0.0191		
Diabetes	1.283 (1.017, 1.618)	0.0358	1.280 (1.007, 1.626)	0.0435	1.951 (1.311, 2.903)	0.001		
Myocardial infarction	1.258 (1.006, 1.572)	0.044			2.272 (1.562, 3.303)	< .0001	1.672 (1.083, 2.582)	0.0203
Left Buddle Branch Block	0.792 (0.605, 1.036)	0.0891			1.413 (0.738, 2.707)	0.297		
PR interval	1.003 (1.001, 1.006)	0.0086			1.003 (0.999, 1.007)	0.1413		
QRS duration	1.001 (0.997, 1.004)	0.7099			1.007 (1.003, 1.011)	0.0016		
LVEF	0.950 (0.933, 0.968)	< .0001	0.951 (0.934, 0.970)	< .0001	0.954 (0.941, 0.968)	< .0001	0.978 (0.961, 0.995)	0.0127
**Therapy**								
Antiarrhythmics excluding beta blockers	1.502 (1.206, 1.870)	0.0003	1.287 (1.016, 1.630)	0.0366	1.777 (1.210, 2.611)	0.0034		
Beta blockers	0.768 (0.604, 0.977)	0.0319			1.050 (0.717, 1.538)	0.7014		
ACEI/ARB	0.662 (0.527, 0.832)	0.0004	0.711 (0.561, 0.901)	0.0048	1.066 (0.736, 1.546)	0.7343	0.555 (0.375, 0.822)	0.0033
Diuretics	1.613 (1.136, 2.292)	0.0076			3.612 (2.396, 5.445)	< 0.0001	2.132 (1.343, 3.384)	0.0013
ICD implanted	0.840 (0.647, 1.090)	0.1901	0.652 (0.495, 0.860)	0.0024	-	-		
CRT-D implanted	0.627 (0.473, 0.832)	0.0012	0.417 (0.308, 0.564)	< .0001	1.669 (1.029, 2.707)	0.038		
**Region**								
Southeast Asia	**1 (ref)**		**1 (ref)**		**1 (ref)**		**1 (ref)**	
East Asia	0.973 (0.779, 1.216)	0.8111	0.561 (0.416, 0.756)	0.0002	0.515 (0.355, 0.748)	0.0005		
South Asia	0.709 (0.541, 0.930)	0.0129			1.341 (0.840, 2.140)	0.2183		
Latin America	1.106 (0.678, 1.805)	0.6856			2.093 (1.122, 3.903)	0.0202	2.014 (1.047, 3.874)	0.036
Europe	0.509 (0.190, 1.365)	0.1795			0.680 (0.168, 2.750)	0.5881		
Middle East and Africa	1.439 (0.808, 2.562)	0.2164			3.769 (2.022, 7.027)	< 0.0001	3.684 (1.876, 7.237)	0.0002

*Abbreviations: ICD* implantable cardioverter-defibrillator, *CRT-D* cardiac resynchronization therapy-defibrillator, *PP* primary prevention, *SP* secondary prevention, *NYHA* New York Heart Association, *ICM* ischaemic cardiomyopathy, *NICM* non-ischaemic cardiomyopathy, *NSVT* non-sustained ventricular tachycardia, *LVEF* left ventricular ejection fraction, *ACEI/ARB* angiotensin-converting enzyme inhibitor/angiotensin receptor blocker

### Improve SCA study and other large primary prevention registries

**Fig. 3:**
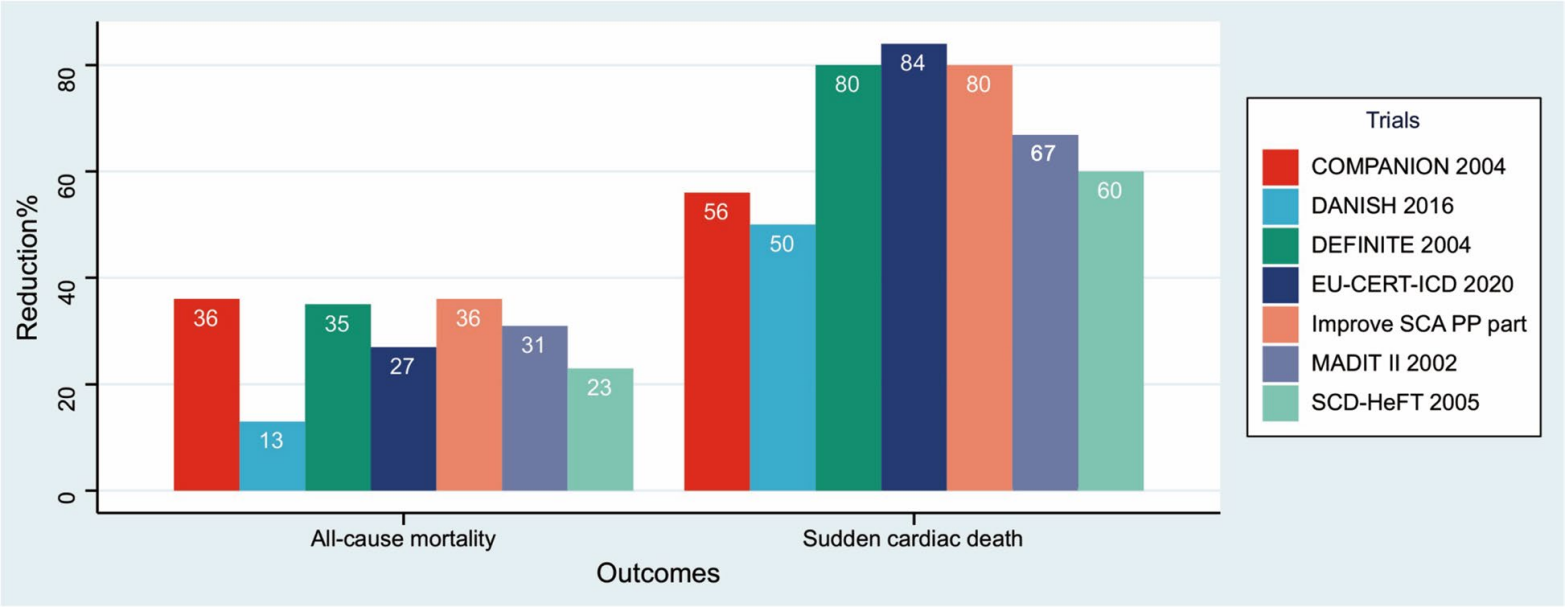
Percentage of all-cause mortality and SCD reduction in ICDs/CRT-Ds primary prevention patients between the Improve SCA and non-Asian-Pacific registries. Abbreviations: ICD, implantable cardioverter-defibrillator; CRT-D, cardiac resynchronization therapy-defibrillator; SCD, sudden cardiac death; SCA, sudden cardiac arrest

Differences in the baseline demographic and clinical characteristics of PP patients between the Improve SCA registry and previous large-scale PP registries from the 2000s are shown in Table 3. Unlike previous studies, the PP cohort from the Improve SCA study included more Asian participants (89.8%) and fewer white participants (6.9%), and the majority came from UMI and LMI countries (71.3%) (Table 3). The use of ACEIs/ARBs was the lowest (71.2%) in the Improve SCA registry. However, the percentage of all-cause mortality reduction, obtained from a proportional hazards regression model incorporating a time-to-event analysis, was 36% (HR = 0.64, 95% CI 0.531–0.802, *p* < 0.0001) for PP patients in the Improve SCA study, which was tied for the highest percentage among all patient cohorts (Fig. 3). Additionally, the percentage reduction in SCD for PP patients in the Improve SCA study was 80% (HR = 0.20, 95% CI = 0.116–0.343, *p* < 0.0001), a value second only to that in the EU-CERT-ICD patient cohort (84%) (Fig. 3).

**Table 3:** Differences in baseline demographic and clinical characteristics in primary prevention patients between the Improve SCA registry and previous large-scale non-Asian-Pacific registries from the 2000s

	**MADIT II 2002 **[8]	**DEFINITE 2004 **[9]	**COMPANION** **2004 **[10, 11]	**SCD-HeFT** **2005 **[12]	**DANISH** **2016 **[13]	**EU-CERT-ICD** **2020 **[14]	**Improve SCA** **2020**
*N*	1232	458	1520	2521	1116	2247	2696
*N*. of patients with defibrillator	742	229	595	829	556	1516	1399
Median follow-up time (months)	20	29	16	45.5	67.6	28.8 ± 13.2	21.6 ± 10.2
Age (years)	64	58.3	66	60	63	62.4	61
Male (%)	84.4	71.2	67	77	72.6	81.8	76.7
NICM (%)	0	100	45	47	100	35	68.5
Country income level LMI/UMI/HI (%)	0/0/100	0/0/100	0/0/100	0/0/100	0/0/100	0/? /Mainly HI	21.2/50.1/28.7
Ethnicity (%)							
Black or African (%)	-	25.8	-	16.9	-	-	0.5
White (%)	87	67.2	Mainly White	76.6	Mainly White	Mainly White	6.9
Asian (%)	-	0.2	-	-	-	-	89.8
**Medical history**							
NYHA Class, I/II/III/IV (%)	36.5/34.6/24.2/4.6	21.6/57.4/21/0	-/-/86/14	-/69/31/-	-/53/45/1	60.5(I/II)/39.5(III/IV)	1.2/47/51.7/0
Hypertension (%)	53	-	-	55.6	31.2	-	38.7
Diabetes (%)	34.98	22.9	41	30.4	19	30.3	30.5
Myocardial infarction (%)	100	0	-	45	0	-	35.1
CRT implanted (%)	0	0	79.7	0	58	0	25.5
NSVT (%)	-	90.6	-	23.1	-	-	29.7
LVEF mean (%)	23.2	21.4	21.2	25	25	28	27
QRS duration (ms)	120	115.1	160	112	146	105.4 ± 17.7	118
LBBB(%)	18.6	19.7	70.8	-	53.6	-	24.1
**Medicine**							
Beta blockers (%)	62	84.9	68	69	92	94.3	75.7
ACEI/ARB (%)	89	96.7	90	94	97	91.2	71.2
Diuretics (%)	75.6	86.7	> 94	82	-	72.2	84

*Abbreviations: NICM* non-ischaemic cardiomyopathy, *LMI* lower middle income, *UMI* upper middle income, *HI* high income, *ICD* implantable cardioverter-defibrillator, *CRT* cardiac resynchronization therapy, *NYHA* New York Heart Association, *NSVT* non-sustained ventricular tachycardia, *LVEF* left ventricular ejection fraction, *LBBB* left Buddle Branch Block, *ACEI/ARB* angiotensin-converting enzyme inhibitor/angiotensin receptor blocker

## Discussion

The Improve SCA registry is the first international registry to study patients with high SCD risk in emerging countries. The key results of this subanalysis of the Improve SCA registry were as follows: (1) There was significant heterogeneity among patients with high SCD risk in emerging countries. The influence of geographic region on patient characteristics, therapy utilization and clinical outcomes in terms of mortality and SCD was significant. (2) In addition to the variables identified in previous studies, geographic region and ICD use were associated with all-cause mortality in a Cox regression analysis, both individually and after accounting for other baseline factors. (3) Primary prophylactic ICDs/CRT-Ds implantation was associated with 36% lower all-cause mortality risk and an 80% lower SCD risk in the Improve SCA registry, which appeared to be higher than that in most previous landmark PP studies.

Our results revealed striking geographic variations in clinical characteristics, therapy utilization and outcomes among patients. The percentage of PP and SP patients without ICDs/CRT-Ds was highest in South Asia. Patients from South Asia had the lowest rate of ICDs/CRT-Ds implantation and the highest rate of SCD. These results are consistent with the Asian SCD in Heart Failure (ASIAN-HF) registry [15, 16]. In the present study, patients from South Asia also had the highest rates of myocardial infarction and the lowest usage rates of ACEIs/ARBs, which may also contribute to the high rate of SCD independent of the low rate of ICDs/CRT-Ds implantation (see also ^14^). An important difference between these two patient cohorts was that the patients enrolled in the Improve SCA met the class I indication for ICDs/CRT-Ds implantation according to ACC/AHA/HRS or ESC guideline recommendations, while patients enrolled in the ASIAN-HF study had HF and an LVEF < 40% or ≥ 50%, and it was assumed that all ICD-eligible patients were implanted for PP [16, 17]. Patients from Southeast Asia had the highest comorbidity burden of hypertension and diabetes, the lowest LVEF and the least ACEI/ARB use. These are plausible reasons why the rate of all-cause mortality was highest among PP patients in Southeast Asia [18]. These results are also consistent with the ASIAN-HF registry study; when compared with patients from South and East Asia, patients from Southeast Asia had the highest rate of comorbidities and all-cause mortality as well [15].

Multivariate Cox regression modelling demonstrated that ICD implantation was a protective factor for all-cause mortality (HR = 0.652, 95% CI = 0.495–0.860, *p* = 0.0024) in PP patients. As such, our data confirmed the utility of the ICDs for the PP of SCD, which had been demonstrated by previous landmark trials [8–14, 19]. Unlike previous studies, these first prospective multinational data from emerging countries highlight the important influence of geographic region on all-cause mortality. After multivariate-adjusted Cox regression analysis, assuming proportional hazards across time, PP patients from East Asia were associated with all-cause mortality, and SP patients with ICDs/CRT-Ds from Latin America and the Middle East and Africa were associated with all-cause mortality. Therefore, the data suggest that regional variations in all-cause mortality might be due to factors that are not well described or measured in the current literature. These factors may include health-care quality, access, the level of patient education, infrastructure, environmental factors and genetics.

Previous landmark trials have included patient populations with low ethnic diversity and predominantly included patients from HI countries. Ethnic differences in SCD have not been fully explored, and the few studies that have explored the issue have yielded inconsistent results. According to a cohort study of residents in the USA, Asian American patients had a lower risk of SCD than African American and White patients [20]. The limited data may have contributed to the perception that Asian patients may be at lower risk of SCD and thus less likely to benefit or have unknown benefits from ICD therapy. For reasons such as this, it remains important to understand the survival of non-White patients or patients from non-HI countries who receive an ICD for PP in clinical practice. However, there are limited data on this topic. Unlike previous registries, the Improve SCA PP population contained more Asian patients (89.8%) and more patients from UMI and LMI countries (71.3%) (Table 3). Although there was no head-to-head comparison between patients from Asia and North America or Europe, ICDs/CRT-Ds implanted for PP in the Improve SCA registry showed superior trends in reducing the rates of all-cause mortality and SCD. The reduction in all-cause mortality, incorporating time-to-event analysis, was 36% in the Improve SCA PP patients with ICDs/CRT-Ds, which appeared to be higher than those seen in previous studies from the 2000s, which ranged from 13 to 36%^17–23^. The reduction in the SCD rate was 80% among PP-implanted patients in the Improve SCA study, which was a rate lower only than that in the EU-CERT-ICD registry (84%) [14]. The reduction rates of all-cause mortality and SCD were similar to those of previous single trials conducted in emerging countries [21]. Therefore, our study with more Asian patients and more patients from UMI/LMI countries prospectively confirmed the benefit found in previous studies for prophylaxis PP patients. There may be several explanations for this. The simplest explanation is that better medical treatment reduces the risk of death. Guideline-directed medical therapy such as ACEIs/ARBs, angiotensin receptor neprilysin inhibition and beta blocker could reduce all-cause mortality and SCD as demonstrated by previous studies [18, 22–24]. However, the use of beta blocker was lower in the Improve SCA study than in those studies conducted after 2010. And the use of ACEIs/ARBs was lowest in the Improve SCA study when compared with previous studies, and the protective effect of ACEIs/ARBs was demonstrated after multivariate adjustment in this study and previous work [18, 22, 23]. These data also suggest that efforts to improve the uptake of guideline-directed medical therapy in emerging countries are warranted. Another reason may be that most patients in this study were from UMI/LMI countries. It has been previously demonstrated that the incidence of SCD is relatively high in geographic areas with lower socioeconomic status [25] and that the all-cause mortality rate among patients from UMI/LMI countries was higher than that among patients from HI countries [26]. Last but not least, patients from UMI/LMI countries had lower implantation rates of ICDs/CRT-Ds when compared with those from HI countries. Implantation of ICDs/CRT-Ds has already been demonstrated to significantly reduce the rates of all-cause mortality and SCD^17−23^. These data may help emerging countries expand their reimbursement guidelines to include ICDs/CRT-Ds for the PP of SCA and convince regulators to provide coverage for the implantation of ICDs/CRT-Ds in PP patients. Although ICDs/CRT-Ds reduce the rate of all-cause mortality and SCD, they are still associated with risk for long-term complications such as lead dislodgement, lead failure and endocarditis [27, 28]. Therefore, large-scale and long follow-up clinical trials with head-to-head comparisons may still be needed.

### Limitations

The study had some limitations. First of all, SP patients who did not receive an ICD/CRT-D (10.6% of SP patients and 3.2% of all patients) were not followed, so the SCD rate among SP patients may be underestimated. Second, deaths were not adjudicated by a central committee; as such, the likelihood of inconsistencies in the classification of deaths must be taken into consideration. Third, due to the limited availability of information pertaining to other variables, such as the education levels of patients, medical care of the country, data of wearable cardioverter defibrillator, inherited primary arrhythmia syndromes and others, we could not adjust for certain confounders. Finally, the study did not have a randomized design, as it was designed to reflect real-world clinical practice in understudied regions. To control for potential bias, the mortality analysis was adjusted to account for patient characteristics that may have a significant effect on mortality.

## Conclusions

These first prospective multinational data from emerging countries highlight the significant heterogeneity among patients with a high risk of SCD in these countries and the important influence of geographical region on patient characteristics and outcomes. Independent predictors of all-cause mortality identified in this study may aid in the risk-stratified targeting of limited resources for SCD prevention in emerging countries. Finally, in view of the benefits of ICDs/CRT-Ds implantation in PP patients in emerging countries, improvement in the implantation rate of ICDs/CRT-Ds is extremely urgent.

### Supplementary Information

**Additional file 1: Table S1.** Baseline demographic and clinical characteristics by country income level.

## Data Availability

The datasets used and/or analysed during the current study are available from the corresponding author upon reasonable request.

## Abbreviations

ACC/AHA/HRS: American College of Cardiology/American Heart Association/Heart Rhythm Society
ACEI/ARB: Angiotensin-converting enzyme inhibitors/angiotensin II receptor blockers
CRT-D: Cardiac resynchronization therapy-defibrillator
ESC: European Society of Cardiology
HI: High income
ICD: Implantable cardioverter-defibrillators
ICM: Ischaemic cardiomyopathy
LMI: Lower middle income
LVEF: Left ventricular ejection fraction
NYHA: New York Heart Association
PP: Primary prevention
SCD: Sudden cardiac death
SCA: Sudden cardiac arrest
SP: Secondary prevention
UMI: Upper middle income
