# Supplementary material for: Regional disparities and risk factors of mortality among patients at high risk of sudden cardiac death in emerging countries: a nonrandomized controlled trial
Source: BMC Med. 2024 Mar 22;22:130. doi: 10.1186/s12916-024-03310-5 (PMC10960430; doi:10.1186/s12916-024-03310-5)
Supplement: Supplementary file 1 — Additional file 1: Table S1. Baseline demographic and clinical characteristics by country income level. [file 12916_2024_3310_MOESM1_ESM.docx]

**Additional file 1: Table S1.** **Baseline demographic and clinical characteristics by country income level**

| **Characteristics** | **LMI** | **UMI** | **HI** | **P value** |
| --- | --- | --- | --- | --- |
| **N** | 825 (21.2%) | 1948 (50.1%) | 1116 (28.7%) | <0.0001 |
| **Demographics** |  |  |  |  |
| Age (years) | 56.3±12.5 | 59.8±12.9 | 59.6±14.2 | <.0001 |
| Male | 664 (80.5%) | 1445 (74.2%) | 872 (78.1%) | 0.0006 |
| **Indication for ICD/CRT-D** |  |  |  |  |
| PP | 646 (78.3%) | 1375 (70.6%) | 675 (60.5%) | <0.0001 |
| ICD/CRT-D implant | 300 (36.4%) | 1313 (70.6%) | 852 (76.3%) | <0.0001 |
| CRT-D implanted | 93 (11.3%) | 542 (27.8%) | 176 (15.8%) | <0.0001 |
| **Medical History** |  |  |  |  |
| NYHA Class, I/II/III/IV (%) | 4.1/68.2/23.4/0.5 | 4.6/28.7/59.1/1.5 | 6.8/50.6/28.8/0.8 | <0.0001 |
| ICM | 228 (27.6%) | 365 (18.7%) | 245 (22.0%) | <0.0001 |
| NICM | 387 (46.9%) | 1279 (65.7%) | 572 (51.3%) | <0.0001 |
| Congestive heart failure | 125 (15.2%) | 692 (35.5%) | 506 (45.3%) | <0.0001 |
| Syncope or presyncope | 125 (15.2%) | 398 (20.4%) | 149 (13.4%) | <0.0001 |
| NSVT | 98 (11.9%) | 679 (34.9%) | 209 (18.7%) | <0.0001 |
| Hypertension | 258 (31.3%) | 647 (33.2%) | 531 (47.6%) | <0.0001 |
| Diabetes | 324 (41.0%) | 408 (21.1%) | 334 (29.9%) | <0.0001 |
| Myocardial infarction | 426 (51.6%) | 462 (23.7%) | 372 (33.3%) | <0.0001 |
| Left Buddle Branch Block | 169 (20.5%) | 421 (21.6%) | 145 (13.0%) | <0.0001 |
| PR duration (ms) | 164.1±38.1 | 176.8±41.4 | 180.0±41.4 | <0.0001 |
| QRS duration (ms) | 119.5±35.0 | 123.7±34.7 | 119.5±32.9 | 0.0009 |
| LVEF (%) | 28.3±8.0 | 32.2±12.5 | 32.9±14.7 | <0.0001 |
| **Baseline Therapy** |  |  |  |  |
| Antiarrhythmics, excluding β blockers | 265 (32.1%) | 1018 (52.3%) | 377 (33.8%) | <.0001 |
| β blockers | 635 (77.0%) | 1487 (76.3%) | 650 (58.2%) | <.0001 |
| ACEI/ARB | 502 (60.8%) | 1298 (66.6%) | 667 (59.8%) | 0.0002 |
| Diuretics | 618 (74.9%) | 1502 (77.1%) | 673 (60.3%) | <.0001 |
| **Outcomes** |  |  |  |  |
| All-cause mortality (PP) | 70 (10.8%) | 183 (13.3%) | 64 (9.5%) | 0.0289 |
| All-cause mortality (SP) | 23 (12.8%) | 49 (8.6%) | 40 (9.1%) | 0.2186 |
| Sudden cardiac death (PP) | 46 (7.1%) | 36 (2.6%) | 6 (0.9%) | <0.0001 |
| Sudden cardiac death (SP) | 15 (8.4%) | 10 (1.7%) | 4 (0.9%) | <0.0001 |

Abbreviations: LMI, lower middle income; UMI, upper middle income; HI, high income; ICD, implantable cardioverter-defibrillator; CRT-D, cardiac resynchronization therapy-defibrillator; PP, primary prevention; SP, secondary prevention; NYHA, New York Heart Association; ICM Ischemic cardiomyopathy; NICM, Non-ischemic cardiomyopathy; NSVT, non-sustained ventricular tachycardia; LVEF, left ventricular ejection fraction; ACEI/ARB, angiotensin-converting enzyme inhibitor/angiotensin receptor blocker.
